# Italian Public Health Response to the COVID-19 Pandemic: Case Report from the Field, Insights and Challenges for the Department of Prevention

**DOI:** 10.3390/ijerph17103666

**Published:** 2020-05-22

**Authors:** Emanuele Torri, Luca Gino Sbrogiò, Enrico Di Rosa, Sandro Cinquetti, Fausto Francia, Antonio Ferro

**Affiliations:** 1 Department of Health and Social Policies, Autonomous Province of Trento, 38122 Trento, Italy; 2 Department of Prevention, Local Health Authority 3 of Venice, 30174 Veneto Region, Italy; lucagino.sbrogio@aulss3.veneto.it; 3 Department of Prevention, Local Health Authority 1 of Rome, 00135 Lazio Region, Italy; enrico.dirosa@aslroma1.it; 4 Department of Prevention, Local Health Authority 2 of Treviso, 31100 Veneto Region, Italy; sandro.cinquetti@aulss2.veneto.it; 5 Department of Prevention, Local Health Authority of Bologna, 40124 Emilia Romagna Region, Italy; fausto.francia@ausl.bologna.it; 6 Department of Prevention, Local Health Authority of Trento, Autonomous Province of Trento, 38123 Trento, Italy; antonio.ferro@apss.tn.it

**Keywords:** COVID-19, pandemic response, case report, Italy, hygiene and public health, public health department

## Abstract

The coronavirus disease (COVID-19) outbreak is rapidly progressing globally, and Italy, as one of the main pandemic hotspots, may provide some hard lessons for other countries. In this paper, we summarize the current organizational capacity and provide a pragmatic and narrative account of strategies and activities implemented by the Department of Prevention (Dipartimento di Prevenzione)—the regional entity of the Local Health Authority of the Italian National Health Service in charge of public health—since the beginning of the outbreak. We conduct a preliminary analysis of general strengths, weaknesses, opportunities, and threats (SWOT) of the response strategies from a local perspective. Furthermore, we provide firsthand insights on future directions and priorities to manage this unprecedented pandemic. Our case report gives a qualitative view of the healthcare response, based on the experience of frontline professionals, with the aim to generate hypotheses about factors which may promote or hinder the prevention and management of a pandemic locally. We highlight the importance of a public health approach for responding to COVID-19 and reshaping healthcare systems.

## 1. Background

The 2019–2020 severe acute respiratory syndrome coronavirus 2 (SARS-CoV-2) outbreak, which was first identified in Wuhan, Hubei, China, in December 2019, has resulted in an ongoing coronavirus disease (COVID-19) pandemic. Exponential increases in the number of confirmed COVID-19 cases and deaths continue to be reported globally, and necessitate awareness and proactive action [1,2]. The infection spread rapidly within China and across countries, with cases in Europe initially limited to small clusters in Germany, France, and the United Kingdom [3].

On 30 January 2020, the World Health Organization (WHO) declared the COVID-19 outbreak a public health emergency of international concern, and on the same day, the Italian government banned air traffic from China [4,5]. The following day, the Italian government declared a six-month state of health emergency and entrusted to the Civil Protection Department the responsibility for the coordination of interventions necessary to deal with the emergency in the national territory [6].

In Italy, only three cases were identified at the end of January, and all involved individuals who had recently traveled to China. The first case of pneumonia due to SARS-CoV-2, without history of possible exposure abroad, was diagnosed in northern Italy’s Lombardy on 20 February [7]. Within a few days, several COVID-19 cases were confirmed in the surrounding areas, and they included a substantial number of critically ill patients. Based on the number of cases and the advanced disease stage of the disease, it was estimated that community spread had occurred since January 2020 [7]. Following this unexpected finding, extensive contact tracing and testing of close contacts unveiled ongoing transmission in several municipalities of the Lombardy region [8]. Another cluster of patients with COVID-19 was simultaneously identified in Veneto, which abuts Lombardy [7]. The first death linked to COVID-19 occurred on 21 February in the municipality of Vo’ (Padua Province) [9].

On February 21, the Ministry of Health ordered a mandatory supervised quarantine for 14 days for all individuals who had come into close contact with confirmed cases of disease. In addition, it required a mandatory communication to the Department of Prevention—the entity of the Local Health Authority of the Italian National Health Service in charge of public health—in identified areas from anyone who had entered Italy from areas with a high risk of COVID-19, followed by quarantine and active surveillance [5]. The Italian government imposed increasingly stringent physical distancing measures, starting with the closure of 10 municipalities in the Lodi province (Lombardy) and one in the Padua province (Veneto) on 23 February 2020 [7]. The same day, the Ministry of Health suspended all public events or facilities of any nature open to the public in five regions in northern Italy (e.g., schools, gyms, public places) [4,5].

Thereafter, additional social distancing measures were initially enforced in Lombardy and within areas of other affected regions. Subsequently, draconian lockdown measures were extended nationwide, through several decrees enacted by the President of the Council of Ministers, closing schools (5 March), banning public events (9 March), limiting movement of people except if motivated by proven work needs or situation of necessity (11 March, national lockdown ordered), and suspending all retailing and business activities, with the exception of essential goods and production activities that were strategic or relevant to the management of the crisis (22 March) [4,9,10,11,12,13,14]. The national lockdown, with exception for some retailing and business activities, was prolonged until 3 May [15].

In March, the number of cases identified in Italy had rapidly increased, predominantly in northern Italy, although all regions of the country reported ever-increasing numbers of COVID-19 cases [16,17]. The WHO Director-General declared COVID-19 a global pandemic on 11 March 2020 and called on governments to take “urgent and aggressive action” to change the course of the outbreak [18]. Thus far, Italy remains one of the global hotspots of active COVID-19 cases with a high fatality rate [19,20,21]. From February to mid-March, the epidemic rapidly progressed towards a scenario of widespread community transmission in the country. The continued increase in SARS-CoV-2 transmission has consequently placed tremendous pressure on the healthcare system and overburdened hospitals in Lombardy and some other areas of central Northern Italy [22,23,24]. However, since April, a response to the abovementioned proactive measures has become evident, with the flattening of the contagion curve trajectory and a reduction in the number of new cases, with a consequent alleviation of the pressure on hospitals and intensive care wards [6,19].

Nevertheless, despite the de-escalation measures that are underway, the situation is far from normal and the Italian National Health Service (INHS; Servizio Sanitario Nazionale) has faced the worst threat since its establishment more than 40 years ago. From the beginning of the crisis, professionals of the Italian healthcare system employed in all its different settings (i.e., public health, primary care, community care, emergency services, hospital care, pharmacies) have been fully and strenuously engaged in battling the SARS-CoV-2 outbreak. In this context, the Italian public health community has been at the forefront, fire-fighting this sweeping pandemic.

With this case report, we aim to provide a general and qualitative perspective on the pandemic response implemented by the Department of Prevention (Dipartimento di Prevenzione, DP), which is the operative branch of the local health authorities of the INHS in charge of public health. Drawing from the hard lessons of COVID-19 in Italy, the Western country most affected by the pandemic to date, we share a preliminary epicrisis and reflections on the current situation, as well as insights and priorities for the future. Firstly, the institutional role and activities of DPs during the initial phase of the pandemic are set out. Secondly, strengths and weaknesses, threats, and opportunities are described. Thirdly, we generate hypotheses about factors which may promote or hinder the prevention and management of a pandemic locally.

## 2. Context and Role of the Department of Prevention

Italy’s healthcare system comprises a regionally organized NHS. The fundamental principles of the INHS include universal coverage, social financing from general tax revenues, and non-discriminatory access to healthcare services. The system provides largely free healthcare at point of delivery, and is regionally administered, with the central government sharing responsibility with the respective regions for healthcare services planning and delivery [25]. At present, Italy ranks 6th globally in terms of highest life expectancy [26]. In 2016, healthcare spending in Italy accounted for 8.94% of gross domestic product (GDP), lower than the average 12.59% for the member countries of the Organization for Economic Cooperation and Development [27]. The WHO and the Organization for Economic Cooperation and Development, in cooperation with various international entities and by using validated scores, have analyzed the INHS and reported that it has developed considerable strengths over the years, including public health policies and achievements [28,29,30]. However, reasons for concern included systemic issues, such as impact of prolonged cuts and cost-containment measures, widespread shortage of human resources, regional variations in the quality of services and outcomes [25,30,31], ageing workforce (average age of 51 years) [32], delays in the digitization process [33], and inadequate budget allocation for preventive measures [34]. For several years, the Italian Observatory on Prevention has been providing in-depth analysis on organizational capacity, staffing, and competences of the DP across Italian regions and territories [35].

The Ministry of Health is the main institution responsible for public health at the national level. The functioning of the Ministry of Health is supported by several established governmental agencies. At present, the most important such agency for public health, including emergency responses, is the National Institute of Health (Istituto Superiore di Sanità), which undertakes scientific research, surveillance, and promotion of public health practices. Within each region, the responsibility for the organization and delivery of services rests on specific geographically and population-defined institutions—the Local Health Authorities (Aziende Sanitarie Locali). The Department of Prevention, which is one of the three ‘pillars’ of the INHS (legislative decrees no. 502 of 1992 and no. 229 of 1999), is the operative branch of every local health authority in charge of preventive medicine and public health, including pandemic response.

The main functions of Departments of Prevention include prophylaxis of infectious and parasitic diseases; protection from the health risks of living environments and workplaces (e.g., environmental pollutants); veterinary safety and health (including epidemiological surveillance of animal populations and prevention of infectious and parasitic diseases); veterinary pharmacovigilance; hygiene of livestock production, and protection of health and hygiene of food of animal origin); food hygiene and safety; nutritional surveillance and prevention; and health promotion on noncommunicable diseases [30]. Departments of Prevention have organizational and financial autonomy. The DP staff include public health physicians and other specialists (i.e., occupational medicine physicians), veterinarians, health assistants, environmental and workplace prevention technicians, and other professionals such as nurses, biologists, chemists, and engineers. To fulfil its mission, the DP guarantees comprehensive governance and integration between the different units and activity areas, and is expected to coordinate with the districts (managing within the Local Health Authority the provision of primary care services, including family medicine and community services) and hospitals and other agencies (e.g., Regional Agency for the Environment, Experimental Zooprophylactic Institutes, National Institute for Insurance against Accidents at Work).

## 3. Local Response to the COVID-19 Pandemic

The epidemic scenario changed rapidly, as did the response strategy and measures. From 22 January (circular no. 1997 of the Ministry of Health) to mid-April 2020, the Italian government enacted over 160 decrees, orders, circular letters, notices, advices, or protocols related to the COVID-19 emergency (e.g., criteria for screening, case reporting, quarantine, biosafety) [4]. Additional policies and ordinances (e.g., resource allocation, physical distancing measures, testing procedures) were published by regions and local health authorities and updated on a daily basis. Local health authorities and DPs have had to keep pace with the implementation of updated guidance, especially for case finding and testing, quarantine and home isolation, and public health measures.

We summarize below the practical measures, grouped into three categories that were instituted for the containment and mitigation of the epidemic and were managed directly by the DPs.

(1) Support to the local health authority for coordination and management of the crisis:
  - Membership of local crisis task force.
  - Epidemic risk assessment and analysis of scenarios and health system capacity (e.g., isolation, laboratory testing, and medical services).
  - Development and updating of local emergency plans.
  - Coordination with institutions (i.e., local councils, school authorities, civil protection) and non-healthcare bodies (e.g., business categories).
  - Provision of information and education to healthcare workforce (e.g., use of personal protective devices, hand washing, waste management).
  - Advice for vulnerable groups (e.g., elderly and frail population), schools, and businesses.
  - Designating dedicated facilities for isolation.
(2) Internal reorganization and infrastructure:
  - Contingency plan: rescheduling non-urgent activities and maintaining only essential programs (e.g., childhood vaccination), telemedicine (e.g., legal medicine), assignment of staff to COVID-19 tasks, and protection of the workforce.
  - Joint integrated response with primary care teams for detection, assessment, rapid reporting, and active surveillance of suspected or confirmed COVID-19 cases.
  - Establishment of an alert system and dedicated helpline.
  - Information system for the management and traceability of testing.
  - Sharing practices and data with other DPs.
(3) Blocking and tackling:
  - Active investigation of cases (suspected, probable, and confirmed) and clusters and contact tracing (concentric circles approach).
  - Deciding on the necessity for quarantine and home isolation.
  - Swabbing and testing, especially through proximity diagnostics (i.e., mobile units, drive-through).
  - Active surveillance for people under quarantine and home isolation.
  - Implementation of orders and advice on physical distancing, environmental hygiene, mortuary police, veterinary medicine, and food safety.
  - Guidance and controls on physical distancing and other preventive measures in different community settings (e.g., at grocers, factories, public spaces).
  - Advice and support for risk assessment and health surveillance in workplaces and other community settings (e.g., nursing homes).
  - Risk communication and community engagement practices [36].
  - Travel advice and screenings of travelers.
  - Collection, analysis, and dissemination of surveillance data (i.e., dashboard).

## 4. SWOT Analysis

We undertook a strengths, weaknesses, opportunities, and threats (SWOT) analysis to provide a quick, pragmatic, and preliminary overview of the perceived state of response strategies and operations from the DP perspective. Such a tool has been applied elsewhere for reviewing healthcare response to COVID-19 [37].

We drew from published literature, practical experience, and consensus brought together at short notice by a group of senior members of Italian Society of Hygiene, Preventive Medicine, and Public Health. In March and April 2020, we performed two rounds of discussion linked to two online meetings of members of the extended college of operators of Prevention, Public Health. and Medical Directorates (composed of 25 public health professionals with governance roles within the scientific society) and one additional meeting of preparation of an online webinar on the state of pandemic response in Italian regions. Themes were collected and reviewed by the authors. To generate the SWOT factors, we set a limit of seven items for each factor, and we selected only items with a full consensus among professionals on priority, generalizability, and relevance.

For analyzing and discussing results, we relied on available data from: the Italian Observatory on Prevention (pre-pandemic state of DPs) [35], a societal working group on DP, and official COVID-19 information provided by the National Institute of Health and Italian Civil Protection [16], as well as ongoing published studies on COVID-19 (most are cited in the references).

We summarize below the results of the analyses of strengths, weaknesses, threats and opportunities.

### 4.1. Strengths

- National event-based surveillance and analysis for public health emergencies and laboratory capacity for detecting prioritized diseases and providing real-time data [38].
- Well-functioning national influenza surveillance network that is supported by regional reference laboratories [39].
- Consolidated experience in first-line response to infectious emergencies, including the severe acute respiratory syndrome (SARS), the influenza pandemic, and Chikungunya, as well as the measles outbreaks and epidemic meningitis.
- National coordination and progressive strengthening of the response, consistent with risk assessments and recommendations of the World Health Organization and the European Center for Disease Prevention and Control.
- Key role of DP in the governance of the emergency within the Local Health Authority.
- Comprehensive and integrated community-based response supported by all the units of the DP (i.e., public hygiene, occupational health, food and nutrition, veterinary healthcare, epidemiology).
- Awareness and early activation of the public health community.

### 4.2. Weaknesses

- Inadequacy of national and regional public health emergency preparedness as well as response planning and operations, including laboratory capacity and medical countermeasures [38], which limited the ability to scale up the response.
- Rapid change, perceived inconsistency, and bureaucratic rigmarole (especially initially) in technical orders and clinical protocols (e.g., case investigation and detection, testing criteria, application of physical distancing), sometimes due to international guidance (e.g., testing criteria, public masking).
- Non-homogenous and limited operational capacity of DP and integration with primary care.
- Patchwork responsiveness and communication at DP level, given the time needed to implement an unprecedented organized response.
- Reactive and “local mindset” in critical thinking and “homemade” solutions to support operations of DPs (i.e., contingency plans and organigrams, applications of national guidance).
- Sparse availability of tools for data integration and smart technologies deployable to support contact tracing, surveillance, and other public health interventions.

### 4.3. Opportunities

- Greater mandate and expanded capacity for preparedness planning, surveillance, and scientific advisory support from European and Italian national authorities.
- Rapid upgrade of national and regional infrastructure for emergency response, including increased human resources capacity and “protected” supply chains.
- Allocation of resources and reengineering integration models and tools for robust collaboration, particularly “intra-territorial care”, between public health and primary care agencies.
- Pragmatic and action-oriented reframing of duties and powers between healthcare and other institutional authorities and politics.
- Accountable response supported by exponential technologies (e.g., smartphone applications, ultra-rapid testing, artificial intelligence, networks, and sensors) and effective policies for real-life implementation of telemedicine.

### 4.4. Threats

- Insufficient resilience of supply chains for protective equipment and other medical countermeasures.
- Health consequences of prolonged interruption or delay of population-based prevention programs (e.g., mass oncological screening, vaccination campaigns) and clinical care.
- Inconsistencies between regional strategies that alter the ability to set up uniform, robust planning for future responses to support overextended health and social care.
- Substantial countrywide social, political, and economic disruption that impacts health and social services.

## 5. Lesson Learned

A proactive and targeted public health response is fundamental for interrupting human-to-human transmission chains and preventing further spread, thereby reducing the intensity of the epidemic. Therefore, in response planning, even at the local level, it is crucial that decision makers primarily understand the pandemic as a public health problem and not as a hospital (or intensive care) issue, especially after the emergency phase is over and the necessity for the containment role of hospitals is greatly diminished. The strengths and weaknesses of the local public health response during the first pandemic phase in Italy were several and varied. Support to the local health authority for coordination and management of the crisis, internal reorganization, and infrastructure guaranteed governance and stewardship for public health action. The cooperation with primary care was fundamental for managing “blocking and tackling” activities.

Core fieldwork and labor-intensive practices for contact tracing and quarantining might have been affected by initial delays in tailoring the emergency response, multiple systemwide organizational and technological constraints, and excessive focus on symptomatic people during the epidemic’s peak. In a rapidly changing epidemiologic scenario, it was essential to adapt strategies to locally available capacity and resources (e.g., staffing, laboratory reagents, protective equipment, information systems, communication tools) and pressure on healthcare services.

Until we have effective vaccines and treatments, public health work will continue to be crucial for protecting people and communities.

In the 14th century, starting in 1348 during a bubonic plague outbreak in medieval Italy, physicians and public health officials started to implement some of the world’s first effective practices of anti-contagion measures related to social distancing and disinfection of surfaces [40]. Currently, non-pharmaceuticals interventions are essential tools in the armamentarium for controlling COVID-19, and it appears that Italy and other nations in Europe and worldwide will have to rely on such measures to varying degrees during the immediate future for reducing the burden of COVID-19 [41]. Resources need to be allocated accordingly, and bold and creative responses will be required. Critical investment in public health is required to safeguard wellbeing and avert the personal and financial tolls of future pandemics [42].

Evidence to date from China, South Korea, and other Asian countries (e.g., Taiwan), as well as data emerging from some European countries, indicates that early decisive actions may reduce community transmission [20]. Furthermore, in moving from containment to mitigation, a combination of stringent measures achieved a meaningful reduction in transmission in Italy [3,5,16].

The comparatively high rate of critical cases and deaths observed in Italy versus some other high-income countries could be attributable to: demography and epidemiological factors [7,22,43], low availability of hospital and intensive care beds, and causal contribution of SARS-CoV-2 infection to recorded deaths [22]. The outbreak was concentrated in a few northern Italian cities and provinces that have seen their hospitals crash under the burden [22]. Moreover, in Italy, both contact tracing and laboratory testing were initially more limited than in better-prepared Asian countries (i.e., South Korea, Taiwan), and the lockdown had to be used as a last, blind measure of desperation [22]. However, data-associated factors that are related to varying cases and cumulative deaths across countries and regions globally need to be carefully investigated, comparing similar timeline and contexts to understand the real impacts of COVID-19 and thus inform public health action.

At the time of writing (20 April), in Italy there were almost 81,000 people in home isolation (with over 180,000 active cases of SARS-Covid-2) and around 1,400,000 tests performed (around 23,000 tests per one million population) [16,19]. In Italy, according to an epidemiology model, cumulatively, 5.9 (1.9–15.2) million people may have been infected as of 28 March, giving an attack rate of 9.8% (3.2%–25%) of the population, which was the second highest in Europe [14]. Another recently published study estimates a proportion of infected population of 4%, varying greatly among Italian regions, from 0.35% in Sicily and Basilicata (southern Italy) to 13.35% in Lombardy [5].

Italian regional and local responses were remarkably different. The regional structure of the Italian national health service caused diverse regional responses to the emergency [44].

A thorough scientific comparative analysis of facts and outcomes is needed. Certainly, the COVID-19 crisis exacerbated existing fault lines and vulnerabilities that everyone in the public health community has recognized and warned about for years. What we can argue is that the response was more effective and proactive where organization, qualifications and competencies, operational capacity, and territorial capillary presence of Departments of Prevention were consistent with the current national legislation and guidance for organization, practice, and allocation of resources within the Italian National Health Service. A high level of integration of public health with primary care and hospital services is another factor hindering an effective response.

An interesting Italian case study on the value of a territorial response may be provided by the Veneto Region, which probably benefited from an early but circumscribed outbreak [9]. In this region, proactive case and contact investigation, testing, and quarantine or home isolation, in parallel with systematic home care of mild cases, were the cornerstones of the territorial response strategy that was based on a well-established network of public health and primary care services. Veneto has reported higher rates of coronavirus testing and home isolation and lower rates of hospital admissions and fatalities, in comparison to Lombardy and some other Northern Italy regions impacted by the outbreak [16]. Veneto opted for strict containment of the outbreak and piloted mass testing in selected areas (i.e., 4.4% of the population were tested, compared with 1.8% in the rest of Italy), whereas Lombardy reported high transmission and disease rates and strengthened hospital services to meet a massively increased demand for hospitalization and intensive care unit beds [44]. In addition to unique epidemiology and environmental factors [43], a possibly delayed public health response and insufficient support and integration of hospital services with community and primary care services might have been among reasons explaining why Lombardy has had 113.1 deaths per 100,000 population, six times higher than Veneto (19.2) [44]. According to a recent estimate, the period prevalence of infection in Veneto is 2%, which is, together with the bordering autonomous region of Friuli Venezia Giulia (1.8%), the lowest across central Northern Italian regions [5].

Public health approaches, smart social distancing policies, aggressive tactics for supporting preventive measures in the community (e.g., hand hygiene, physical distancing), active surveillance, contact tracing and testing, and home care as an elective setting for early clinical treatment of mild COVID-19 patients during containment and mitigation of epidemics may reduce the growth of the contagion curve, prevent the saturation in hospital services and nosocomial viral spread, decrease the number of deaths, and enable de-escalation of control measures.

Other lessons relevant to other countries, from the difficult but nonetheless educational episode that Italy has experienced, indicate the need:

- To act swiftly in protecting medical personnel, to avoid loss of personnel capacity and limit nosocomial virus spread.
- For early and smart swabbing primarily linked to epidemiological investigations, and tracing of high-yield contacts (including home testing) and risk categories (e.g., health and social care workforce, personnel of “essential services”).
- For acute respiratory infection/influenza-like illness sentinel surveillance through sentinel general practice and telephone/app helplines.
- For policies and rationale for rapid point-of-care diagnostic testing and seroepidemiologic population surveys.
- For optimizing the use and benefits of public health interventions through real-time accessibility of data and systematic evaluation.

We grounded our assumptions and hypotheses about the factors which promote or hinder the prevention and management of a pandemic locally on perception and data available in an emergency and rapidly changing context. In addition, Italy has a slightly different health system compared to other developed systems, and we are aware that our analysis and suggestions may not be applicable in other settings. SWOT results and discussion relied on a pragmatic exchange and consensus process and were not based on any systematic literature review or quantitative analysis. Therefore, information gathered may be oversimplified, some data overlooked, and authors’ biases introduced. Despite such limitations, we believe this case report can provide a preliminary, contextualized, and comprehensive insight into the local response of the Italian Department of Prevention and the value of certain public health measures during the COVID-19 crisis. Raising awareness of the complexity of activities to perform during an epidemic crisis may help countries and public health officials in their strategic planning and on-the-ground decision making.

## 6. Conclusions

In moving from a strict physical distancing period (Phase 1) to a staged reopening phase (Phase 2), until an effective vaccine or treatment is available (Phase 3), public health is essential in managing risks of increased transmission of COVID-19 to the health and wellbeing of the public, society, and the economy, via measures taken to reduce the spread of the disease [45].

Therefore, after the end of lockdown, in going back to a “new normal”, public health will be the key driver for informing decision-making with the engagement of all stakeholders. Measures to phase out confinement need policies and actions at local, regional, national, and international levels [46]. During Phase 2, the three key pillars for preventing cases and clusters from becoming outbreaks and epidemics will be: (1) proactive surveillance of cases in collaboration with primary care and general practitioners; (2) timely and extensive testing and contact tracing; and (3) evidence-based management of public health measures in the community. Peer-to-peer warning apps being developed in Italy and other countries to support mass contact tracing will be very helpful to scale up testing and tracing capacity, but need to be used within a clear social, legal, and value framework.

While scientific research and biomedical innovators are tackling the virus at unprecedented speed, additional systemwide priorities are:

- Driving and organizing boot-on-the-ground prevention and surveillance to protect vulnerable groups and the general population.
- Continuing to reshape the public health risk assessment and management system to ensure optimal epidemic response capacity.
- Continuing education and capacity building of public health workers.
- Standardizing terminology, communication tools, guidelines, and actionable protocols for practice.
- Replacing structurally bureaucratic management and low-value interventions with pragmatic local, community-based practice and scientifically sound practice.

Hopefully, we will be able to ride the wave of this dramatic health crisis, transforming it into a relevant scientific and professional opportunity for hygiene and public health, building in the post-COVID-19 period a cutting-edge, attractive, and modern discipline [47].

The community of public health physicians, health assistants, and other prevention professionals working in DPs and in other positions within the Italian NHS are fully committed to a continued timely, professional, non-fungible, and resilient action and practice exchange. Sharing experiences in the field, in addition to research, is crucial for the better management of healthcare services during the pandemic crisis for saving lives and protecting communities.

## 7. Key Points

- The Department of Prevention is the entity of the Local Health Authority of the Italian National Health Service in charge of hygiene and public health, including the management of epidemiologic emergencies.
- Since the beginning of the outbreak, the work of the Departments of Prevention was crucial in supporting coordination and management of response operations at the local level, as well as supporting and managing activities of case finding, tracing and testing, and quarantining and home isolation, as well as other public health community measures (e.g., hygiene practice, workplace surveillance).
- Analysis and reporting of the local response may inform understanding of complexity of actions and challenges for controlling the COVID-19 pandemic faced by public health professionals.
- A community-based response to COVID-19 needs public health and primary care; regional and local differences in organization and strategies may explain different outcomes.
- Public health work is a key driver for a safe lifting of social distancing restrictions after the end of the lockdown.
